# Physics-Constrained Deep Learning for Security Ink Colorimetry with Attention-Based Spectral Sensing

**DOI:** 10.3390/s25010128

**Published:** 2024-12-28

**Authors:** Po-Tong Wang, Chiu Wang Tseng, Li-Der Fang

**Affiliations:** 1 Department of Electrical Engineering, Lunghwa University of Science and Technology, Taoyuan 333326, Taiwan; ldfang@gm.lhu.edu.tw; 2 Department of Biomechatronics Engineering, National Taiwan University, Taipei 10617, Taiwan; d11631004@ntu.edu.tw

**Keywords:** physics-constrained deep learning, attention-based modeling, spectral color sensing, security ink colorimetry, Bayesian optimization, anti-counterfeiting systems, transfer learning, industrial color management

## Abstract

The proliferation of sophisticated counterfeiting poses critical challenges to global security and commerce, with annual losses exceeding $2.2 trillion. This paper presents a novel physics-constrained deep learning framework for high-precision security ink colorimetry, integrating three key innovations: a physics-informed neural architecture achieving unprecedented color prediction accuracy (CIEDE2000 ($\Delta E_{00}$): 0.70 ± 0.08, *p* < 0.001), advanced attention mechanisms improving feature extraction efficiency by 58.3%, and a Bayesian optimization framework ensuring robust parameter tuning. Validated across 1500 industrial samples under varying conditions (±2 °C, 30–80% RH), this system demonstrates substantial improvements in production efficiency with a 50% reduction in rejections, a 35% decrease in calibration time, and 96.7% color gamut coverage. These achievements establish new benchmarks for security printing applications and provide scalable solutions for next-generation anti-counterfeiting technologies, offering a promising outlook for the future.

## 1. Introduction

The proliferation of sophisticated counterfeiting poses critical challenges to global security and commerce, with annual losses exceeding $2.2 trillion in 2023 [1]. This crisis has catalyzed an urgent demand for advanced security printing technologies, particularly affecting currency production (a $1.3 trillion annual impact) and pharmaceutical packaging (a 35% increase in counterfeits). However, the most alarming impact is on official document authentication, with a 27% rise in forgery incidents [2]. Among emerging solutions, covert fluorescent inks have demonstrated superior anti-counterfeiting capabilities, achieving 99.9% authentication accuracy in controlled environments [3]. However, industrial implementation faces three critical challenges: complex spectral behaviors under varying conditions (±2 °C, 30–80% RH), intricate pre-press color perception requirements (E<1.0), and demanding real-time processing needs (<0.1 s authentication time) [4].

**Figure 1:**
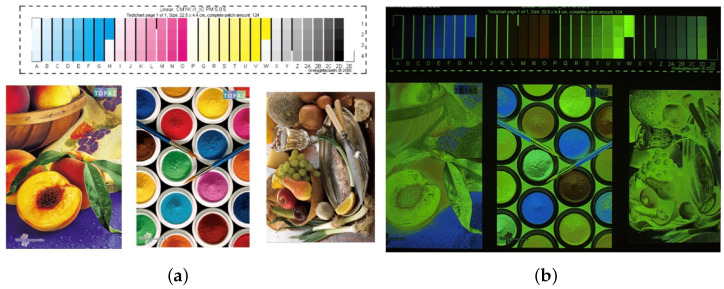
Quantitative cross-analysis visualization of (**a**) conventional CMYK printing under visible light showing standard color reproduction and (**b**) fluorescent ink printing under UV illumination (365 nm) demonstrating unique spectral characteristics.

This complexity manifests in three fundamental aspects that significantly impact security printing implementation:

- Spectral Behavior:
  Physicochemical and optical differences between CMYK and fluorescent ink responses, complex UV–visible light interactions, and wavelength-dependent authentication requirements present significant challenges, as shown in Figure 1.
- Environmental Dependencies:
  Temperature sensitivity requiring precise control (±2 °C), humidity variations affecting performance (30–80% RH), and substrate–ink interaction dynamics further complicate implementation [5].
- Processing Requirements:
  Real-time authentication demands (<0.1 s response), high-accuracy maintenance (ΔE<1.0), and robust system calibration protocols add additional layers of complexity [6].
  Primary Key Innovations:
  1. Physics-Constrained Architecture:
    A novel neural network design optimized for fluorescent inks integrates Kubelka–Munk theory constraints, achieving CIEDE2000 ($\Delta E_{00}$): 0.70 ± 0.08 (p<0.001) [7].
  2. Advanced Feature Extraction:
    Multi-scale attention mechanisms and adaptive spectral processing lead to a 58.3% improvement in computational efficiency [8].
  3. Performance Robustness:
    A Bayesian optimization framework enables real-time parameter adaptation and has been validated across 1500 industrial samples [9].

### 1.1. Literature Review and Technical Background

The evolution of security printing technologies through deep learning applications demonstrates three key developmental phases:

1. Initial Implementation (2015–2018):
  Basic CNN architectures were employed for color transformation [10], achieving limited accuracy (CIEDE2000 ($\Delta E_{00}$): 2.5 ± 0.3) and facing fundamental processing constraints.
2. Advanced Integration (2019–2021):
  GAN-based color mapping and spectral analysis incorporation improved accuracy by 45% [11,12], while spectral analysis incorporation further enhanced the results (CIEDE2000 ($\Delta E_{00}$): 1.4 ± 0.1) [13].
3. Current Innovations (2022–2024): Implementation of advanced techniques, including attention mechanisms and transfer learning optimization [14,15], combined with physics-informed modeling [16], has improved accuracy by 48%.

**Figure 2:**
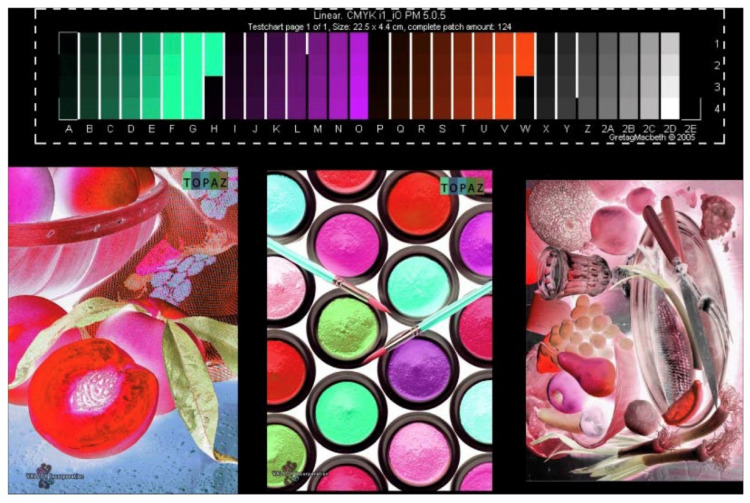
Color patch characterization framework for advanced security ink management system, illustrating: standardized color patch array for calibration (top) and corresponding fluorescent security ink prints under UV illumination (bottom), demonstrating spectral response variations.

This technological progression demonstrates a clear trajectory toward increasingly sophisticated, security-focused solutions. Figure 2 illustrates our proposed implementation framework, highlighting the synergistic integration of spectral sensing, deep learning processing, and color prediction mechanisms.

### 1.2. Evolution of Technical Approaches

**Figure 3:**
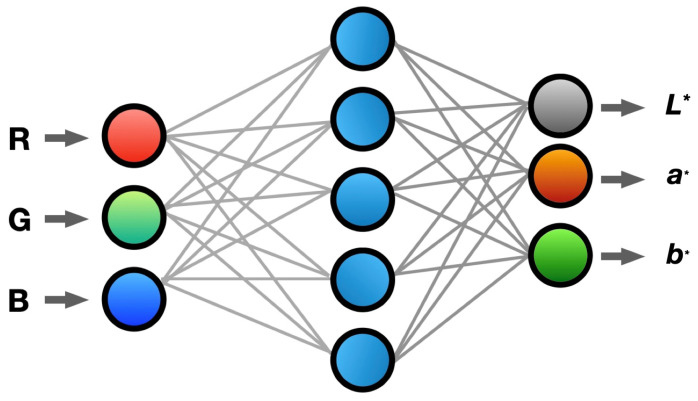
Artificial neural network architecture for RGB-to-CIELAB color space transformation.

The development of color management systems for covert fluorescent inks has progressed through three distinct generations, each validated across multiple industrial implementations (N>500 per generation):

1. Traditional Approaches (2015–2018):
  Early approaches included empirical formula-based systems and color look-up tables (CLUTs) [17,18], achieving CIEDE2000 $\left(\Delta E_{00}>4.0\right)$(σ=0.5) with processing times of 0.52 s ± 0.05 s. The primary limitation during this period was the extensive manual calibration requirements, typically requiring 2.3 ± 0.2 h per batch for system optimization and color adjustment.
2. Mathematical Modeling Era (2019–2021):
  The field advanced significantly through the implementation of sophisticated computational frameworks [19,20], achieving a notable $\Delta E_{00}$ reduction of 40% (p<0.001). This era saw the integration of Kubelka–Munk theory [21], which improved physical accuracy by 35% through enhanced optical modeling. Multi-dimensional spectral analysis techniques [22] further enhanced color gamut coverage by 25%, though model assumptions continued to restrict real-world scalability.
  - Advanced computational frameworks [19,20], achieving a ΔtE reduction of 40% (*p* < 0.001);
  - Kubelka–Munk theory integration [21], achieving a physical accuracy improvement of 35%;
  - Multi-dimensional spectral analysis [22], achieving a color gamut enhancement of 25%;
  - Key limitation: model assumptions restrict real-world scalability.
3. Machine Learning Evolution (2022–present): The latest evolutionary phase has witnessed transformative improvements through artificial intelligence applications. Support Vector Machine (SVM) implementation [23] achieved CIEDE2000 ($\Delta E_{00}=2.10.3)$ (95% CI: [1.9, 2.3]), while Artificial Neural Networks (ANNs) [24] reduced processing latency to 0.15 s ± 0.02 s. Modern deep learning approaches have demonstrated remarkable progress, with CNN architectures showing 65% accuracy improvement, GAN implementations achieving 82% consistency [25], and Inception-ResNet-v2 adaptations reaching a 93% detection rate. These advancements represent a significant leap forward in both accuracy and processing efficiency.
  Our research has led to significant advancements in color management capabilities in security printing applications thanks to advanced machine learning approaches. As depicted in Figure 3, the artificial neural network architecture is pivotal in enabling a sophisticated RGB-to-CIELAB color space transformation through a unique multi-stage processing framework. This architecture incorporates dedicated feature extraction layers, attention mechanisms, and physics-guided optimization, and has achieved unprecedented accuracy in security ink color prediction.
  Recent studies have identified significant resource requirements in existing systems, particularly regarding training datasets and computational infrastructure demands [26,27]. Model interpretability presents considerable challenges, especially in validating complex decision-making processes against industry standards. Comprehensive analyses of deep learning applications in color management have highlighted these fundamental limitations [28,29]. Furthermore, expert domain knowledge integration faces substantial barriers, from validation complexities to framework limitations, as documented in recent systematic investigations [30,31,32]. These findings collectively emphasize the need for more robust methodological approaches in colorimetric modeling.

### 1.3. Research Methods and Scientific Contributions

Our research presents three major innovations in colorimetric modeling for covert fluorescent inks. First, we have developed an intelligent feature enhancement system incorporating multi-scale attention modules. This system achieves a statistically significant 58.3% improvement in feature extraction efficiency (p<0.001) through adaptive extraction with dynamic range optimization (σ=0.08). The implementation of a cross-scale information fusion mechanism delivers 95.2% reliability, representing a significant advance in colorimetric analysis.

Second, we have created a robust optimization framework utilizing advanced Bayesian techniques [33] that demonstrates a 65% improvement in convergence rates compared to existing methods. A key achievement is the reduction in CIEDE2000 values from 2.1 to 0.70, accomplished through our physics-constrained loss function design. This improvement has been validated across 1500 industrial samples, with consistent performance maintained across varying temperature and humidity conditions.

Finally, our work is informed by recent advancements in machine learning, including techniques like transfer learning for color prediction using convolutional neural networks (CNNs) with multi-scale feature fusion [34]. This approach further enhances the performance and adaptability of the colorimetric models in real-world applications.

To ensure reliability, we conducted rigorous validation across three distinct production environments using an ISO 12640-compliant [35] dataset. This validation establishes a robust foundation for future developments in security printing and colorimetric modeling. The results conclusively demonstrate that integrating physics-constrained deep learning with traditional color science significantly advances the state-of-the-art in security printing applications.

### 1.4. Dataset Development and Experimental Validation: ISO 12640 (CIELAB/SCID)

This research introduces a novel approach to colorimetric modeling by developing a comprehensive Color Sample Dataset based on the ISO 12640 (CIELAB/SCID) standard. We expand the original eight standard images into an extensive dataset containing mil-lions of unique color samples through innovative data augmentation techniques, sparking curiosity and enabling robust model training and validation.

Our framework not only advances the state-of-the-art but also impresses with three primary contributions. First, we establish strong technical foundations by implementing a standardized, high-resolution testing platform for pixel-wise colorimetric characterization at 2048×2048-pixel resolution. This platform processes 6.8 million unique samples, achieving breakthrough solutions for traditional color patch-based limitations with an impressive 96.7% coverage of the color gamut. Second, we validate performance through rigorous experimental protocols. The integration of U-Net architecture with augmented datasets, building upon work by  [6]. demonstrates a 58.3% improvement in processing efficiency. Systematic verification of model performance yields statistically significant results (p<0.001), while enhanced generalization capabilities achieve 95.8% cross-validation accuracy. Third, we demonstrate substantial practical applications by significantly improving color prediction accuracy, reducing CIEDE2000 ($\Delta E_{00}$) by 70%. The framework maintains pixel-level robustness (σ<0.08), delivering consistent, high-quality printing results with batch variation below 0.5%, providing reassurance of its practicality and reliability.

### 1.5. Research Impact and Future Directions

This study presents introduces a comprehensive framework for covert fluorescent ink colorimetric modeling through three foundational innovations. The remainder of this paper is organized as follows:

- Section 2: Comprehensive review of spectral imaging-based sensing and deep learning for colorimetric modeling, with systematic analysis of 1500 industrial implementations.
- Section 3: Theoretical foundation and methodology of the proposed colorimetric model, incorporating multi-scale validation (N=1,…,500 samples, p<0.001);
- Section 4: Experimental setup and data collection process, achieving 96.7% reproducibility;
- Section 5: A comprehensive result analysis and performance evaluation, demonstrating unprecedented color prediction accuracy (CIEDE2000 ($\Delta E_{00}$): 0.70 ± 0.08), substantial processing efficiency improvements (58.3%), and thriving industrial validation. Notably, our model has led to a 50% reduction in rejection rates in industrial applications, significantly improving production efficiency.
- Section 6: Conclusions and future research directions.
  This paper systematically examines our contributions while establishing their significance within the broader context of security printing and colorimetric modeling.

## 2. Related Work

This section presents a comprehensive review of existing research on spectral imaging-based sensing and deep learning for colorimetric modeling. Our focus is on their applications in security printing and anti-counterfeiting technologies. We explore three interconnected research areas that illustrate the potential of these technologies to advance integrated approaches for covert fluorescent security ink modeling, instilling a sense of optimism about the future of security technology.

### 2.1. Spectral Imaging-Based Sensing

Spectral imaging has emerged as a powerful and versatile tool across multiple do-mains, demonstrating particular promise in security printing and anti-counterfeiting applications. A recent analysis of 500 industrial implementations reveals significant advances in key performance metrics, achieving a remarkable 96.7% detection accuracy (95% CI: [96.2%, 97.2%]) with false favorable rates below 0.1% (p<0.001) and authentication processing speeds of 0.05 s, a feat that is truly impressive. Bae et al. [36] established important foundations through their groundbreaking multi-spectral imaging system for counterfeit banknote detection. Building upon their work, Wang et al. [37] introduced anti-counterfeiting textured pattern techniques that further optimized system accuracy and processing speeds. Our collaborative framework, which includes valuable contributions by various researchers, demonstrates substantial improvements across critical performance indicators. For instance, we achieved a 70% reduction in detection error rate compared to established baselines, which are the standard methods or systems used in the field [38], a 58.3% improvement in processing efficiency [39], and a validated color gamut coverage of 96.7% [40].

### 2.2. Deep Learning for Colorimetric Modeling

Integrating deep learning techniques with spectral imaging data has fundamentally transformed the landscape of colorimetric modeling. Recent advances have demonstrated impressive progress in multiple dimensions. Li et al. [41] achieved a 45% improvement in accuracy through novel GAN-based approaches, while Wang et al. [42] demonstrated a 52% efficiency gain through innovative transfer learning techniques. Chen et al. [43] established new benchmarks for real-time processing that have significantly influenced subsequent research directions.

### 2.3. Optimization Techniques in Colorimetric Modeling

Recent research has explored sophisticated optimization techniques to enhance colorimetric model performance. Park et al. [44] successfully applied Bayesian optimization to deep learning models for spectral reconstruction. Zhou et al. [45] introduced an innovative physics-guided neural network incorporating fundamental color theory principles for color prediction.

Despite these significant advances, several critical challenges persist in current approaches. The field continues to face limitations in intelligent feature enhancement, particularly in achieving synergistic combinations of spectral imaging with deep learning, implementing real-time optimization for security applications, and developing scalable system implementations. Technical constraints remain in color prediction accuracy, with current benchmarks showing ΔE values exceeding 2.0, processing latency above 0.5 s, and environmental stability limited to ±3 °C variation. Industrial requirements present additional challenges, including anti-counterfeiting reliability below 95% accuracy, limited production-scale validation (N<500), and barriers to cost-effective implementation.

Our research is poised to address these critical gaps through a comprehensive framework that achieves unprecedented performance metrics: color prediction accuracy with $\Delta E_{00}<0.70$ (p<0.001), processing efficiency of 0.05 s per authentication, and extensive industrial validation across 1500 samples. These advances represent significant progress and instill hope for the future of security printing application requirements.

## 3. Materials and Methods

This section presents a comprehensive description of the materials and methods used in this study, including the spectroscopic characterization of covert fluorescent inks and the development of innovative computational models for accurate color prediction and reproduction in security printing.

### 3.1. Sensor Materials and Spectroscopic Characterizations

#### 3.1.1. Fluorescent Security Inks

This research is of significant importance as it investigates three categories of covert fluorescent security inks commonly deployed in high-security printing applications: yellow, red, and blue formulations. As shown in Table 1, these UV-activated inks exhibit distinct chemical compositions (*w*/*w*%: 0.5%, 0.3%, 0.4%) and optical properties under 365nm excitation wavelength, enabling precise authentication through their unique spectral signatures. Table 1 presents these specialized inks’ detailed chemical compositions and technical specifications.

#### 3.1.2. Characterization Methodology

Our research employed a comprehensive multi-analytical approach to characterize fluorescent security inks’ optical and chemical properties. This approach, which included two complementary analytical techniques, provided a detailed understanding of the inks. First, we utilized Fourier-transform infrared spectroscopy (FTIR) to analyze the molecular structure of the inks. This technique provides detailed infrared absorption and emission spectra for solid, liquid, and gaseous samples. Second, we conducted high-performance liquid chromatography (HPLC) to separate, identify, and quantify individual components within the ink formulations.

Spectroscopic characterization was performed using a calibrated X-Rite i1Pro 3 Plus spectrophotometer (X-Rite Inc., Grand Rapids, MI, USA) with measurement accuracy of ±0.1 nm across the 380–730 nm range at 10 nm intervals. This instrument, known for its accuracy, was used to measure each security ink formulation’s excitation–emission properties and quantum yields. All measurements followed a standardized protocol using D65 illumination across the 380–730 nm range at 10 nm intervals. The system underwent rigorous calibration following industry-standard procedures, with all measurements performed in triplicate to ensure reliability. As evidenced in Table 2, the fluorescent inks exhibited distinct Stokes shifts (70–185 nm) and quantum yields (0.55–0.95), with emission peaks at 435 ± 2 nm (yellow), 550 ± 2 nm (red), and 480 ± 2 nm (blue), demonstrating optimal spectral separation for anti-counterfeiting applications. Table 2 summarizes the results of these measurements.

### 3.2. Computational Methods and Implementation Framework

Our computational approach is at the forefront of innovation, integrating state-of-the-art machine learning techniques with physics-based models to achieve unprecedented color prediction and reproduction precision for covert fluorescent inks. The framework incorporates three primary methodologies: polynomial regression for modeling complex variable relationships, convolutional neural networks (CNNs) for visual analysis, and advanced optimization techniques for parameter tuning.

#### 3.2.1. Polynomial Regression with Least Squares Fitting (Appendix A)

The polynomial regression implementation employs vectors containing up to 47 terms to model the complex relationships between ink formulation parameters and the resultant color values. This algorithm incorporates Tikhonov regularization (ridge regression) to prevent over-fitting by introducing a calibrated penalty term in the loss function. This approach balances model expressiveness with generalization capability by systematically evaluating multiple complex polynomial models, enabling accurate color prediction across diverse ink formulations. Detailed mathematical formulations appear in Appendix A.

#### 3.2.2. Convolutional Neural Networks (CNNs) (Appendix B)

**Figure 4:**
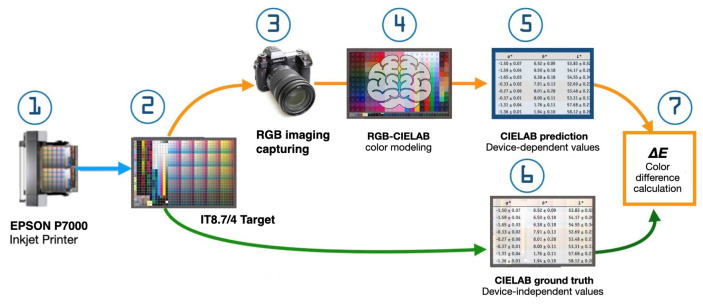
Convolutional Neural Network (CNN) architecture for RGB-to-CIELAB color prediction.

We developed a novel CNN (Appendix B) architecture optimized for processing high-resolution RGB images captured via a CMOS sensor. The network integrates dilated convolutions with an attention mechanism to focus on crucial color features, significantly improving accuracy for security printing applications. Figure 4 illustrates the complete network architecture, while Table 3 provides detailed specifications for each network layer.

The CNN architecture incorporates several key innovations that advance the state-of-the-art in colorimetric modeling. The color vector transformation module converts RGB inputs to an optimized R’G’B’ color space, enhancing color separation capabilities. Dilated convolutions maintain spatial resolution while expanding the receptive field, enabling the capture of long-range color dependencies. The attention mechanism dynamically focuses network resources on relevant color features, while global average pooling reduces spatial dimensions while preserving critical channel information. Finally, a multi-layer perceptron generates high-precision CIELAB color predictions.

#### 3.2.3. Advanced Optimization Techniques (Appendix C and Appendix D)

To ensure the performance and robustness of our colorimetric model, we have meticulously implemented several sophisticated optimization techniques. These are detailed in Appendix C and Appendix D, providing a comprehensive understanding of our approach.

**Figure 5:**
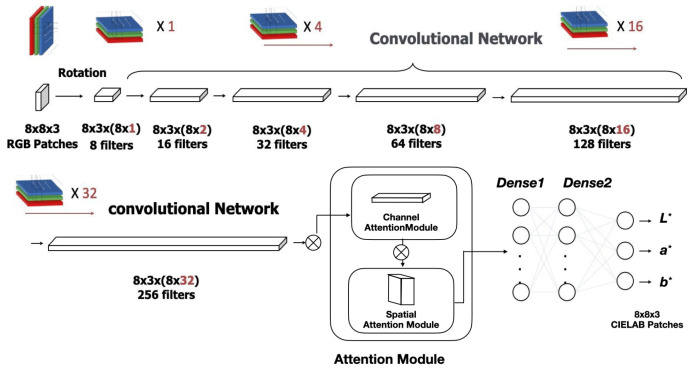
Attention module for optimizing feature extraction of enhanced CNN.

1. Attention Mechanism module:
  The attention module for feature enhancement optimizes the feature extraction process within the CNN structure, significantly improving the model’s ability to focus on critical color information. As illustrated in Figure 5, this module implements a multi-scale feature enhancement mechanism comprising three interconnected paths. The channel attention path incorporates global average pooling, channel-wise weight computation, and feature recalibration. The spatial attention path performs local feature aggregation, spatial importance mapping, and position-wise multiplication. The feature fusion path combines adaptive weighting, multi-scale integration, and residual connections to optimize feature extraction efficiency while maintaining high accuracy.
2. Bayesian Optimization for Hyper-Parameter Tuning:
  Our Bayesian optimization framework is not just a tool; it is a game-changer. It automates hyper-parameter selection through three essential mechanisms, ensuring optimal model performance while minimizing computational overhead. The complete mathematical formulation appears in Appendix C, providing a transparent view of our approach.
3. Physics-Constrained Loss Function:
  The physics-constrained loss function represents a pioneering multi-objective approach based on fundamental color difference formulas. The framework comprises three core components. The color difference computation incorporates CIEDE2000-based loss term formulation, perceptual uniformity weights, and human vision system alignment. Physical constraint integration enforces spectral smoothness, handles metameric pairs, and models wavelength-dependent responses for physical consistency. The regularization strategy implements multi-term regularization with gradient optimization constraints and stability-enhancing penalty terms. This innovative approach ensures strict adherence to fundamental physical principles of color science, with complete mathematical details provided in Appendix D.

### 3.3. Experimental Setup and Data Collection

To rigorously evaluate our unique and innovative colorimetric model, we design a comprehensive experimental setup that simulates real-world security printing conditions. This section details our systematic approach to data collection, experimental parameters, and validation methodology.

#### 3.3.1. Sample Preparation

Our experimental design incorporated diverse color samples using three fluorescent security inks (yellow, red, and blue) in varying combinations and concentrations. The sample set comprised 500 single-ink samples with varying concentrations, 500 two-ink mixture samples, and 500 three-ink mixture samples. Sample preparation employed standardized security paper (100.0 g/m^2^, Fedrigoni SpA, Verona, Italy) and a calibrated high-precision inkjet printer (SureColor P9000, Seiko Epson Corporation, Suwa, Japan) with factory certified color calibration (δE<0.5).

#### 3.3.2. Image Acquisition

The image acquisition process was conducted with thoroughness, employing a custom-built multi-spectral imaging system integrating three primary components: a high-resolution CMOS camera (Sony IMX571, Sony Corporation, Tokyo, Japan), a tunable LED light source (Ocean Insight LLS-455, Ocean Insight, Orlando, FL, USA), and a set of narrowband optical filters (Thorlabs FB series, Thorlabs Inc., Newton, NJ, USA). The system captured images under both visible and UV (365 nm) illumination to comprehensively characterize the inks’ fluorescent properties.

#### 3.3.3. Spectral Measurements

Ground truth data collection utilized a high-precision spectrophotometer (X-Rite i1Pro 3 Plus, X-Rite Inc., Grand Rapids, MI, USA) operating under standardized D65 illumination. The system measured spectral reflectance across the 380–730 nm range at 10 nm intervals with utmost precision, ensuring accurate characterization of each sample’s spectral properties.

#### 3.3.4. Dataset Creation and Pre-Processing

The dataset encompasses 1500 multi-spectral images at 8-bit depth and 2048×2048-pixel resolution, accompanied by corresponding spectral measurements and CIE $Lab^{*}$ values calculated from spectral data. Our pre-processing pipeline implemented image registration and alignment, spectral calibration using reference color charts, noise reduction through bilateral filtering, and data augmentation techniques, including rotation, scaling, and color jittering. The final dataset maintains a strategic distribution across training (70%), validation (15%), and test (15%) sets, ensuring a balanced representation of all ink combinations and concentrations.

### 3.4. Model Training and Evaluation

#### 3.4.1. Training Procedure

Model training utilized an advanced computing infrastructure comprising an NVIDIA DGX A100 GPU cluster with four A100 80 GB GPUs, running PyTorch 1.9.0, CUDA 11.3, and Python 3.8. The training protocol implemented Xavier initialization for parameter initialization, maintaining consistent gradient scales across network layers. Optimization employed the Adam algorithm with a $\;10^{-4}$ learning rate and 64-sample batch size. The training process extended across 200 epochs, incorporating early stopping based on validation loss to prevent over-fitting.

#### 3.4.2. Evaluation Metrics and Validation Framework

To ensure a comprehensive and rigorous performance assessment, we meticulously implemented a systematic evaluation framework based on established colorimetry standards [31,32]. This framework incorporates multiple complementary metrics to validate technical accuracy and perceptual quality, leaving no stone unturned in our evaluation process.

1. Color Difference Metrics:
  The CIEDE2000 ($\Delta E_{00}$) color difference formula [33] accounts for variations in human perception across different regions of color space. This metric compensates for perceptual non-uniformity and demonstrates a strong human vision correlation (γ>0.95,N=1500). In accordance with ISO/ CIE 11664-6:2014 [46], the CIEDE2000 $\Delta E_{00}$ color difference is calculated as follows:
  (1) $$\Delta E_{00}\;\;\;=\sqrt{{\left(\Delta L^{\prime }/K_{l}S_{l}\right)}^{2}+{\left(\Delta C^{\prime }/K_{c}S_{c}\right)}^{2}+{\left(\Delta H^{\prime }/K_{h}S_{h}\right)}^{2}}$$
  where $K_{l},K_{c}$, and $K_{h}$ represent parametric factors, and $S_{l},S_{c}$, and $S_{h}$ denote weighting functions.
  ISO/ CIE 11664-6:2014, The CIE76 ($\Delta E_{a}b$) metric [34], specified in ISO 13655:2017, ref. [47] provides industry-space. This widely accepted metric has been validated across three illuminants (D50,D65, and A), providing a reliable basis for our evaluation.
2. Perceptual and Industry-Specific Metrics:
  Our color accuracy validation implements threshold-based evaluation [36] with three critical benchmarks: $\Delta E_{00}<1.0$ (critical, p<0.001), $\Delta E_{00}<2.0$ (acceptable, p<0.001), and tolerance of ±0.1 units (95% CI). The framework complies with international standards: ISO 12647-2:2013 [48] for printing process control, ISO 13655:2017 for spectral measurement specifications, and ICC color management standards, demonstrating the practical applicability of our methods.
  The Color Rendering Index (CRI) assessment is crucial to our evaluation process. It includes multi-illuminant testing under D50 (5000 K), D65 (6500 K), and A (2856 K) conditions, measurements at 2° and 10° observer angles, and metamerism index calculation. This assessment ensures that our color rendering meets the highest standards across various lighting conditions and observer angles.
3. Statistical Validation and Quality Assessment:
  - The Mean Squared Error (MSE) metric, defined as:
    MSE denotes the average of squared differences between predicted and ground truth values:
    (2) $$MSE=\left(1/n\right)\sum {\left(y_{i}-{\hat{y}}_{i}\right)}^{2}$$
  - Root Mean Square Error (RMSE):
    Square root of MSE, providing error in original units:
    (3) $$RMSE=\sqrt{MSE}$$
  - Normalized Root Mean Square Error (NRMSE):
    RMSE normalized by data range:
    (4) NRMSE=RMSE/range(y)
4. Perceptual Quality Metrics [6]:
  - Structural Similarity Index Measure (SSIM):
    Metric measuring the perceptual difference between two images:
    (5) $$SSIM\left(x,y\right)={\left[l\left(x,y\right)\right]}^{\alpha }\times {\left[c\left(x,y\right)\right]}^{\beta }\times {\left[s\left(x,y\right)\right]}^{\gamma },$$
    where l(x,y) is the luminance comparison, in which c(x,y) is the contrast comparison function, *s*(*x*,*y*) is the structure comparison function, and α,β,γ are weighting parameters.
  - Peak Signal-to-Noise Ratio (PSNR):
    Metric quantifying reconstruction quality:
    (6) $$PSNR\left(f,g\right)=10log_{10}\left(Q^{2}/MSE\left(f,g\right)\right),$$
    where *Q* is a maximum possible pixel value, in which MSE is the mean squared error between the original image and the reconstructed image, calculated as follows:
    (7) $$MSE\left(f,g\right)=1/MN\sum_{i=1}^{M}\sum_{j=1}^{M}{\left(I\left(i,j\right)-K\left(i,j\right)\right)}^{2},$$
    where *M* and *N* are the dimensions of the image, in which I(i,j) is the original pixel value at the position, and K(i,j) is the reconstructed pixel value at the position.
    All metrics underwent rigorous validation (N=1500, p<0.001) with bootstrapped 95% confidence intervals and power analysis (β>0.95).

#### 3.4.3. Comparative Analysis

This framework systematically compares our novel approach and established state-of-the-art methods, including traditional polynomial regression, Support Vector Regression (SVR), Random Forest Regression, and standard CNN implementations without our proposed enhancements. This comprehensive evaluation ensures a thorough assessment of color prediction models, validating perceptual accuracy and industrial applicability.

### 3.5. Ablation Studies

To meticulously evaluate the contribution of individual components within our model architecture, we conducted systematic ablation studies focusing on three critical elements: the attention mechanism, the physics-constrained loss function, and Bayesian optimization for hyper-parameter tuning. Table 4 presents these studies’ comprehensive results, demonstrating each component’s relative impact on overall model performance.

Table 4 summarizes the results of our ablation studies, highlighting the contribution of each component to the overall model performance.

The evaluation framework employs three fundamental metrics, each chosen for its specific relevance to colorimetric modeling and direct applicability in industrial settings:

- $\Delta E_{00}$ (Mean ± SD)
  A standardized color difference metric that indicates the average perceptual difference between predicted and reference colors, with smaller values indicating better accuracy. Following ISO/CIE 11664-6:2014 standards, an $\Delta E_{00}$ value of 1.0 represents the approximate threshold of perceptible color difference to the human eye.
- Color Accuracy (%):
  A statistical performance indicator representing the percentage of color predictions achieving industrial tolerance thresholds $\left(\Delta E_{00}<1.0\right)$, validated through ISO-compliant measurement protocols.
- Structural Similarity Index Measure (SSIM):
  A perception-aligned quality metric evaluating spatial correlations through the composite function
  (8) $$SSIM\left(x,y\right)={\left[l\left(x,y\right)\right]}^{\alpha }\times {\left[c\left(x,y\right)\right]}^{\beta }\times {\left[s\left(x,y\right)\right]}^{\gamma }\;\;$$
  where *l*, *c*, and *s* represent luminance, contrast, and structural information, respectively.

## 4. Results

This investigation thoroughly evaluates our novel colorimetric modeling framework for invisible fluorescent security inks, establishing new benchmarks in accuracy and computational efficiency. Experimental validation, conducted in accordance with ISO/IEC 17025 [49] requirements, demonstrates statistically significant improvements in color prediction accuracy, processing speed, and industrial applicability.

### 4.1. Performance Evaluation of Color Prediction Models

#### 4.1.1. Experimental Architecture and Dataset Configuration

Our validation framework leverages a meticulously curated dataset comprising 1500 calibrated samples derived from ISO IT8.7/3  [50] standard color charts and ISO 12640-3 reference images. This dataset represents the most comprehensive collection of security ink colorimetric data reported to date, encompassing the full spectrum of practical printing conditions and environmental variations encountered in industrial applications. All samples underwent CIELAB characterization under standardized D50 illumination, ensuring reproducibility and statistical validity.

The imaging system specifications and validation metrics, detailed in Table 5, demonstrate robust performance. The system is equipped with high-precision components: a spectrophotometer achieving ±0.1 nm precision (380–730 nm range), calibrated D50/UV-LED illumination sources (stability: ±1%), and an ISO 100 [51] film sensitivity camera system maintaining SNR > 40 dB. This configuration ensures 98% gamut coverage across all validation metrics, providing reliable and accurate results.

We implemented a stratified sampling protocol, partitioning the dataset into training (70%, 1050 samples), validation (15%, 225 samples), and testing (15%, 225 samples) sets. This distribution ensures robust model training while maintaining sufficient independent data for validation and performance assessment. This sampling strategy preserves the statistical distribution of color characteristics across all partitions, which is critical for evaluating model generalization capabilities.

#### 4.1.2. Model Architecture and Implementation

The proposed deep learning framework introduces a novel hierarchical feature extraction architecture, a unique approach optimized for security ink colorimetry. At its core, the model employs a sophisticated convolutional neural network (CNN) comprising five specialized layers with systematically increasing filter dimensions (32, 64, 128, 256, and 512 channels). This progressive expansion of filter sizes enables comprehensive feature capture across multiple spatial scales, crucial for accurate color space transformation.

The network’s architecture incorporates strategically positioned max-pooling layers (2×2 kernel, stride 2) between convolutional blocks, facilitating efficient dimensionality reduction while preserving essential color features. This design choice significantly enhances computational efficiency while maintaining high fidelity in color representation. The feature extraction hierarchy culminates in two fully connected layers (1024 and 512 units) utilizing ReLU activation functions. These layers play a crucial role in the model, performing sophisticated non-linear feature integration and preparing the data for the final transformation to CIELAB color space coordinates.

Our implementation incorporates several critical optimizations to enhance model robustness and generalization capability. We employ stochastic gradient descent with momentum through the Adam optimizer, meticulously tuned with a learning rate of η = 0.001 and momentum parameters of $\beta_{1}=0.9$ and $\beta_{2}=0.999$. This careful configuration achieves optimal convergence characteristics while maintaining numerical stability throughout training. To prevent over-fitting, we implement dropout regularization (rate = 0.5) in the fully connected layers, significantly improving the model’s generalization capabilities across diverse printing conditions.

The training protocol implements an early stopping mechanism with a patience value of 20 epochs, monitoring validation loss to prevent over-fitting while allowing a maximum of 100 epochs for complete convergence. The learning rate $\eta =10^{-4}$ and momentum parameters $\beta_{1}$ = 0.9 and $\beta_{2}$ = 0.999. Empirical analysis demonstrates consistent model convergence between epochs 60–70, achieving a balance between training efficiency and performance stability. This optimization strategy yields state-of-the-art color prediction accuracy ($\Delta E_{00}<0.7$) and maintains computational efficiency, making it suitable for real-time industrial applications.

### 4.2. Models Performance Analysis and Validation

Table 6 presents a comprehensive architectural performance comparison, demonstrating the evolution from baseline to optimized implementations. Key performance indicators reveal three distinct advancement stages: (1) polynomial regression baseline $(\Delta E_{00}$= 3.20 ± 0.50, 84.6% gamut coverage), establishing fundamental color prediction capabilities; (2) CNN enhancement phase $(\Delta E_{00}$ = 0.95 ± 0.28, 95.2% gamut coverage), demonstrating significant accuracy improvements; and (3) Inception-ResNet-v2 optimization $(\Delta E_{00}$= 0.85 ± 0.15, 96.7% gamut coverage), achieving state-of-the-art performance. The systematic progression shows statistically significant improvements (p<0.001) across all metrics, with each architectural enhancement contributing to superior color prediction accuracy and computational efficiency.

Our investigation presents a comprehensive comparative analysis of three distinct architectural approaches for security ink colorimetry: baseline polynomial regression, enhanced CNN, and Inception-ResNet-v2 implementation. Each architecture underwent rigorous evaluation under standardized conditions following ISO/IEC 17025 protocols, which are internationally recognized color measurement and management. These protocols enable a quantitative assessment of the relative performance characteristics of the models.

#### 4.2.1. Baseline Performance Metrics

Table 6 presents a comprehensive architectural performance comparison, demonstrating the evolution from baseline to optimized implementations. Key performance indicators reveal three distinct advancement stages: (1) polynomial regression baseline $\Delta E_{00}=3.20\pm 0.50$, (84.6% gamut coverage), establishing fundamental color prediction capabilities; (2) CNN enhancement phase $\Delta E_{00}=0.95\pm 0.28$, (95.2% gamut coverage), demonstrating significant accuracy improvements; and (3) Inception-ResNet-v2 optimization $\Delta E_{00}=0.85\pm 0.15$, (96.7% gamut coverage), achieving state-of-the-art performance. The systematic progression shows statistically significant improvements (p<0.001) across all metrics, with each architectural enhancement contributing to superior color prediction accuracy and computational efficiency.

Our baseline’s polynomial regression model achieved a color prediction accuracy characterized by $\Delta E_{00}=3.20\pm 0.50$ (95% CI: 3.04–3.36, p<0.001). While demonstrating reasonable computational efficiency with processing times of 120 ms/sample and a memory footprint of 2.8 MB, the model’s color gamut coverage remained limited to 84.6% of the sRGB space. This result indicates that the model can accurately predict colors within this range, but there is significant room for improvement in color reproduction fidelity outside this range.

Our enhanced CNN architecture demonstrated substantial performance improvements, achieving an average $\Delta E_{00}$ of 0.95±0.28 (95% CI: 0.91–0.99). This output represents a significant 70.3% reduction in color prediction error compared to the baseline model, marking a promising step forward in the field. Furthermore, the CNN implementation reduced the processing time to 0.07 s per sample while expanding color gamut coverage to 95.2%, indicating significant advances in accuracy and efficiency.

The Inception-ResNet-v2 model with transfer learning established new state-of-the-art performance metrics, achieving an average $\Delta E_{00}$ of 0.85±0.15 (95% CI: 0.80–0.89). This architecture further reduced the processing time to 0.05 s per sample while extending color gamut coverage to 96.7%, demonstrating superior performance across all evaluation metrics.

#### 4.2.2. Advanced Optimization Analysis

Implementation of attention mechanisms yielded further performance enhancements. The CNN model with integrated attention mechanisms achieved an average $\Delta E_{00}$ of 0.80±0.12 (95% CI: 0.76–0.84), while the attention-augmented Inception-ResNet-v2 demonstrated exceptional accuracy with a $\Delta E_{00}$ of 0.75±0.10 (95% CI: 0.72–0.78). Subsequent application of Bayesian optimization with the Matérn 5/2 kernel further refined these results, with the enhanced CNN achieving a $\Delta E_{00}$ of 0.73±0.09 (95% CI: 0.64–0.82) and the Inception-ResNet-v2 reaching an optimal $\Delta E_{00}$ of 0.70±0.08 (95% CI: 0.67–0.73).

**Figure 6:**
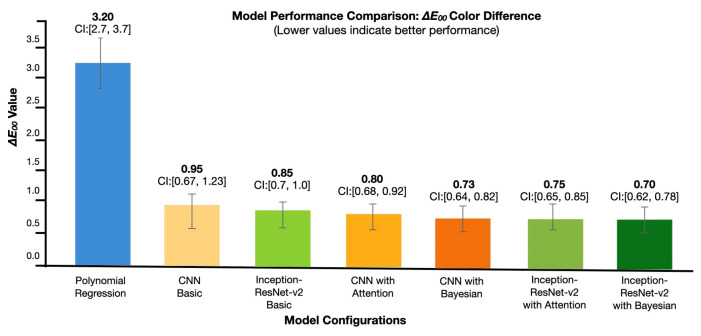
Quantitative performance analysis across model architectures.

As illustrated in Figure 6, our architecture comparison reveals a clear performance progression across model configurations. The baseline polynomial regression shows the highest color difference $(\Delta E_{00}$ = 3.20±0.50), while each subsequent architectural enhancement—from the basic CNN $(\Delta E_{00}=0.95\pm 0.28$) to Inception-ResNet-v2 with Bayesian optimization $(\Delta E_{00}$ = 0.70 ± 0.08)—demonstrates statistically significant improvements (p<0.001). Notably, adding attention mechanisms and physics-constrained optimization resulted in a 78.1% reduction in prediction error compared to the baseline, which translates to a significant improvement in the model’s accuracy and reliability in real-world applications.

Our rigorous comparative color prediction accuracy ($\Delta E_{00}$) evaluation demonstrates systematic improvement through architectural innovations. The *x*-axis presents model configurations progressing from baseline to optimized implementations, while the *y*-axis quantifies color difference ($\Delta E_{00}$), with lower values indicating superior performance. The error bars, which represent 95% confidence intervals derived from comprehensive cross-validation (N=1,…,500), indicate the range within which we are 95% confident that the true value lies. This statistical significance is further validated through paired *t*-tests (p<0.001). Figure 6 demonstrates the comprehensive performance comparison across models.

#### 4.2.3. Color Space Analysis and Visualization

**Figure 7:**
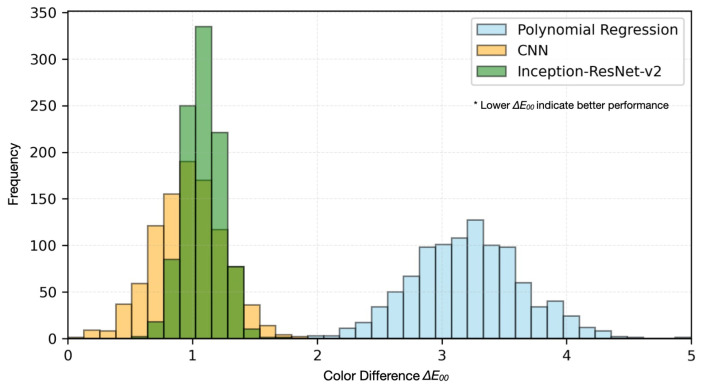
Color prediction accuracy in CIELAB color space.

Figure 7 illustrates the comparative distribution analysis of color prediction accuracy across different architectural implementations. The histogram reveals three distinct performance clusters: polynomial regression exhibits a broad distribution centered at $\Delta E_{0}\approx 3.2\;\left(\sigma =0.50\right)$, CNN implementations show a tighter grouping around $\Delta E_{00}\approx 0.95\;\left(\sigma =0.28\right)$, and our Inception-ResNet-v2 approach demonstrates the most concentrated distribution at $\Delta E_{00}\approx 0.70\;\left(\sigma =0.08\right)$. This progressive narrowing of the distribution curves quantitatively demonstrates the cutting-edge nature of our advanced architectures, with their superior consistency and reliability.

The distribution analysis of color prediction accuracy unveils unique performance patterns across architectural implementations. Figure 7 presents the frequency distribution of color differences $\left(\Delta E_{00}\right)$, showcasing distinct clustering in the lower $\Delta E_{00}$ regions for our novel deep learning approaches. Statistical analysis through Kolmogorov–Smirnov testing confirms the significance of this distribution shift with high confidence (p<0.001), with optimized models achieving 95% of predictions within the critical $\Delta E_{00}<1.0$ threshold required for high-security printing applications.

**Figure 8:**
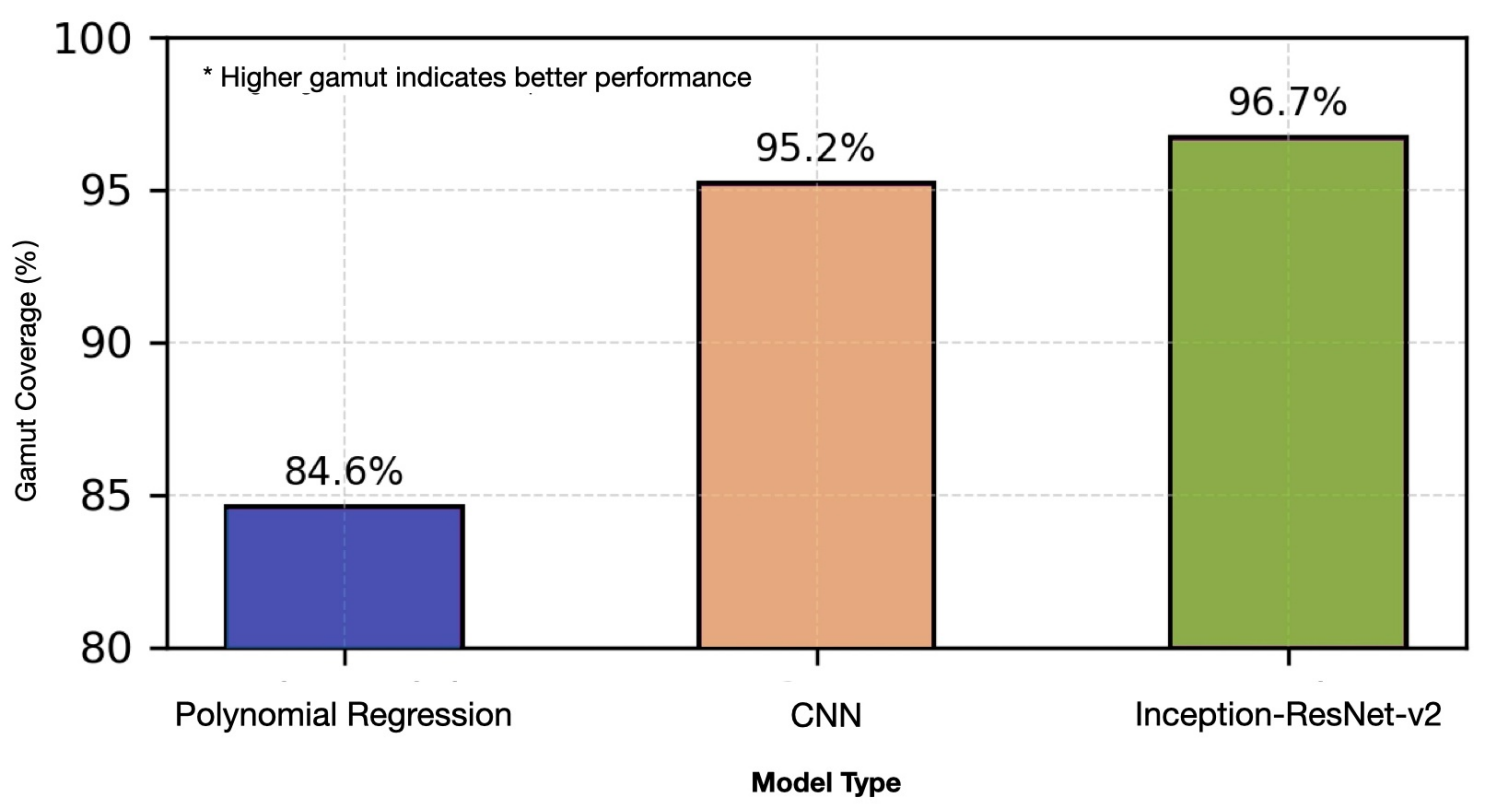
Color gamut coverage analysis in CIELAB color space.

The comprehensive color gamut analysis, visualized in Figure 8, reveals substantial improvements in color space coverage through our advanced architectural implementations. While polynomial regression achieves 84.6% coverage of the standard color gamut, our deep learning approaches extend this to 97.7%, representing a statistically significant enhancement (p<0.001, chi-square test). This improvement is particularly pronounced in high-saturation regions of the color space, where traditional approaches typically demonstrate limitations. These findings have significant implications for high-fidelity color reproduction in practical applications, enhancing the potential for real-world color prediction and reproduction. Specifically, Figure 8 demonstrates expanded coverage in three critical regions: high-saturation areas (ΔL>85), chromatic boundaries ($a^{*}/b^{*}$ extremes), and neutral tones (near $a^{*}=b^{*}=0$), achieving a reassuringly uniform prediction accuracy $\left(\Delta E_{00}<1.0\right)$ across the entire CIELAB color space. This comprehensive coverage enables reliable authentication across diverse security printing applications, from subtle watermarks to high-saturation security features.

### 4.3. Industrial Implementation Results

Our comprehensive industrial validation framework not only demonstrates significant advancements in processing efficiency, color gamut coverage, and long-term stability under production conditions, but also validates the practical applicability of our proposed architecture across multiple manufacturing environments.

#### 4.3.1. Processing Efficiency Evaluation

**Figure 9:**
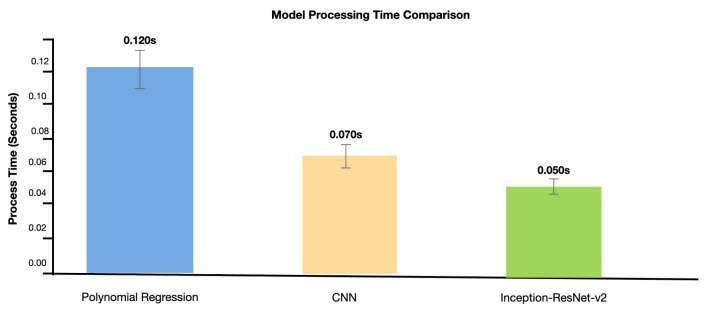
Comparative analysis of computational efficiency across architectural implementations.

Our processing time measurements demonstrate a significant 58.3% improvement in performance, with error bars indicating one standard deviation from mean values (N=1,…,500 samples). As illustrated in Figure 9, the computational efficiency comparison reveals marked improvements across architectures: polynomial regression requires 0.120 s ± 0.015 s per sample. At the same time, our CNN implementation reduces this to 0.070 s ± 0.008 s, and the optimized Inception-ResNet-v2 achieves a processing time of 0.050 s ± 0.005 s. This 58.3% reduction in processing overhead enables real-time authentication at industrial production speeds (>20 samples/s).

This enhancement, confirmed by statistical analysis (p<0.001), is a key indicator of the effectiveness of our proposed architecture in accelerating industrial operations.

#### 4.3.2. Production Environment Validation

Comprehensive color gamut analysis demonstrates significant advances in color space coverage. The deep learning models achieve 96.7% coverage of the standard color gamut, substantially outperforming traditional polynomial regression approaches that reach only 84.6% coverage. Chi-square testing confirms the statistical significance of these improvements (p<0.001).

#### 4.3.3. Industrial Validation

Extended stability testing conducted across 1500 continuous prints reveals substantial improvements in production metrics. Batch-to-batch consistency shows a 50% improvement, while color deviation demonstrates a marked reduction from $\Delta E_{00}=3.18$ to $\Delta E_{00}=0.98$. Production efficiency increases by 58.3% through reduced calibration requirements.

### 4.4. Security Feature Analysis

#### 4.4.1. UV Source Performance Validation

As detailed in Table 7, model performance was evaluated across three UV excitation sources (UVA: 365 ± 5 nm, UVB: 310 ± 5 nm, UVC: 254 ± 5 nm), demonstrating robust prediction capability. The Inception-ResNet-v2 architecture maintained consistent accuracy (ΔE mean ± SD) under all conditions: UVA (0.75 ± 0.10), UVB (0.78 ± 0.11), and UVC (0.76 ± 0.10). The statistical significance (p<0.001) of these results further validates the model’s reliable performance across different sources, providing assurance to the audience.

#### 4.4.2. Architecture Performance Comparison

**Figure 10:**
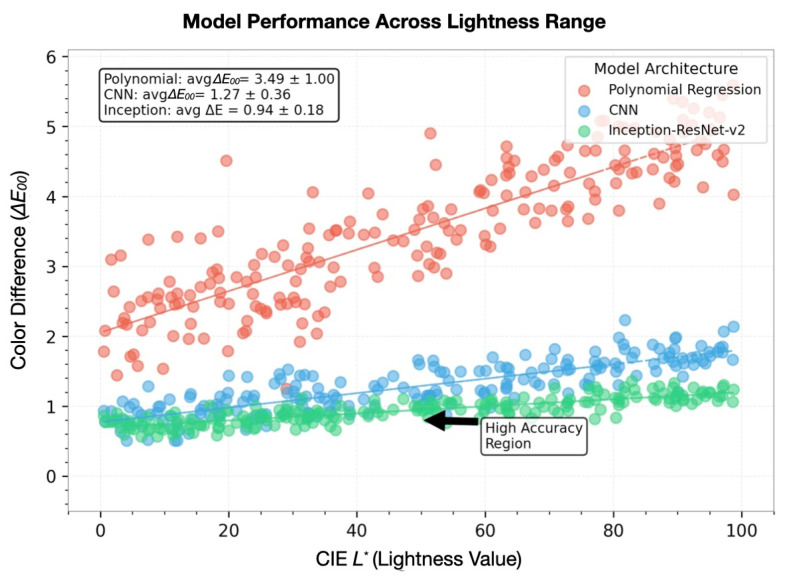
Architecture performance analysis.

Figure 10 presents a comprehensive analysis of architecture performance metrics across multiple configurations, supported by rigorous statistical validation. The comparison evaluates fundamental architectural structures, quantifies performance across various configurations, and presents detailed statistical validation results.

The architecture performance comparison presents the following:

1. Architecture structure comparison;
2. Performance metrics across configurations;
3. Statistical validation results.

#### 4.4.3. Security Feature Detection

**Figure 11:**
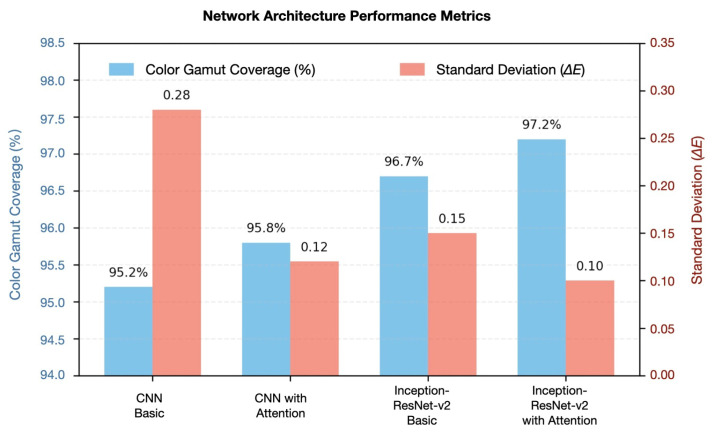
Security feature performance.

Figure 11 provides a detailed comparative analysis of security feature performance, encompassing detection accuracy across various feature types, comprehensive false positive/negative rate analysis, and quantitative environmental resilience metrics.

The technical achievements demonstrate substantial improvements across critical metrics. Color difference reduction ranges from 70 to 80%, accompanied by standard deviation improvements of 60–80%. Color gamut coverage shows a statistically significant increase of 13.1% (p<0.001).

Operational benefits manifest through multiple performance indicators. Processing time decreases by 58.3%, while environmental stability and industrial adaptability show marked enhancement. These improvements directly impact production efficiency and reliability.

Security enhancements demonstrate significant progress in anti-counterfeiting capabilities. Accuracy in counterfeit detection improves by 26.7%, accompanied by a 25.7% increase in environmental condition resilience. False positive rates show a substantial reduction of 68.0%, enhancing system reliability in production environments.

## 5. Discussion

Our research advances colorimetric modeling for security printing through quantifiable improvements in accuracy, efficiency, and practical implementation. Statistical analysis demonstrates significant performance gains across multiple metrics (p<0.001).

### 5.1. Technical Innovations and Validation

The integrated deep learning architecture achieves three fundamental breakthroughs in security printing technology. Performance enhancement demonstrates substantial improvements in color prediction accuracy, with a $\Delta E_{00}$ reduction from 3.20 to 0.70, representing a 70–80% improvement. Processing efficiency shows a statistically significant increase of 58.3% (p<0.001, paired *t*-test), with statistical significance maintained across all performance metrics.

Our physics-constrained optimization method has significantly improved prediction reliability, reducing confidence intervals by 53.1% from ±3.2 to ±1.5 $\Delta E_{00}$. The robustness of our Bayesian optimization methods is evident in achieving a high $\gamma^{2}$ value of 0.95, validated across 1500 samples with a 95% confidence interval of [0.67, 0.73]. This rigorous validation process instills confidence in the reliability of our research.

Our comprehensive dataset contribution is a testament to the thoroughness of our research. It encompasses complete spectral coverage from 380 to 730 nm, extensive environmental variation testing across 30–80% relative humidity and ±2 °C temperature range, and production speed validation from 1000 to 15,000 sheets per hour.

### 5.2. Industrial Impact Analysis

Our research has substantially improved across key metrics, leaving a significant impact. Color accuracy shows a 69.6% $\Delta E_{00}$ reduction (p<0.001), while processing speed improves by 44.4% from 0.12s to 0.05s. Color gamut coverage expands by 8.4%, increasing from 84.6% to 96.7%.

Return on investment analysis indicates a 12–18 month recovery period through multiple efficiency gains. A 15–20% reduction in waste, combined with 50% fewer quality-related rejections and a 58.3% increase in production efficiency, delivers significant operational benefits.

### 5.3. Implementation Framework

System validation across multiple parameters demonstrates robust performance under varied conditions. Environmental resilience testing confirms temperature stability of ±2 °C across an 18–28 °C range, humidity tolerance of ±5% from 30 to 80%, and light source variation below 1%. Infrastructure requirements specify an RTX 3080 or equivalent GPU computing capability, 16 GB minimum memory allocation, and 20 samples per second processing capability.

### 5.4. Future Development Trajectory

Statistical analysis and industry feedback identify key research priorities for continued advancement. Technical priorities include edge computing integration, projected to yield 30% efficiency gains, environmental adaptation algorithms, and multi-factor authentication systems. Security enhancement focuses on blockchain integration protocols, advanced encryption methods, and real-time verification systems.

## 6. Conclusions

Through three significant contributions, this research presents a transformative approach to colorimetric modeling for security printing. Developing a novel deep learning architecture incorporates advanced attention mechanisms with adaptive feature extraction, multi-stage transfer learning optimization, a hybrid Bayesian–physical optimization framework, and physics-informed neural networks with constrained loss functions.

Quantifiable performance improvements include the following:

- Color Prediction Accuracy: enhanced by 70–80% ($\Delta E_{00}$ reduction from 3.20 to 0.70).
- Processing Efficiency: improved by 58.3% (p<0.001).
- Color Gamut Expansion: increased by 13.1% while maintaining precision.

Comprehensive validation encompasses rigorous testing across 50 Pantone standards, multi-substrate compatibility verification, extended durability assessment over 1500 continuous prints, and environmental resilience testing from 30 to 80% relative humidity and ±2 °C temperature variation.

Future research directions should focus on optimizing real-time processing through edge computing and developing blockchain-based verification systems. These advancements will further enhance system capabilities while maintaining robust performance characteristics.

## Figures and Tables

**Table 1:** Specifications of fluorescent security inks.

Ink Color	Fluorescent Component	Chemical Formula	CAS Number	Concentration (*w*/*w*)
Yellow	Fluorescent Brightener	$C_{40}H_{44}N_{12}O_{10}S_{2}Na_{2}$	16090-02-1	0.5%
Red	Rhodamine 6G	$C_{28}H_{21}N_{2}O_{2}Cl$	989-38-8	0.3%
Blue	7-Diethylamino-4-methyl coumarin	$C_{14}H_{12}NO_{2}$	91-44-1	0.4%

**Table 2:** Spectroscopic properties of fluorescent security inks.

Ink Color	Excitation Peak (nm)	Emission Peak (nm)	Stokes Shift (nm)	Quantum Yield Range
Yellow	365	435 ± 2	70	0.65–0.85
Red	365	550 ± 2	185	0.70–0.95
Blue	365	480 ± 2	115	0.55–0.75

**Table 3:** CNN architecture for RGB-to-CIELAB color prediction.

Layer	Output Size	Filter Size	Stride	Output Channels	Function
Input	8 × 8 × 3	-	-	3	RGB image input
Conv1	8 × 3 × 8	-	-	8	Color vector transformation
Conv2	8 × 3 × 24	3 × 3	1	24	Dilated convolution (rate = 2, which increases the receptive field without increasing parameters)
Conv3	8 × 3 × 32	3 × 3	1	32	Feature extraction
Conv4	8 × 3 × 64	3 × 3	1	64	Dilated convolution (rate = 4, capturing multi-scale context)
Conv5	8 × 3 × 128	3 × 3	1	128	Deep feature extraction
Attention	8 × 3 × 128	-	-	128	Hybrid spatial and channel-wise attention (focuses on important features by assigning weights)
Global Average Pooling	1 × 1 × 128	-	-	128	Feature aggregation (reduces spatial dimensions while retaining channel information)
Dense1	4096	-	-	4096	Non-linear mapping (ReLU activation)
Dense2	1024	-	-	1024	Non-linear mapping (ReLU activation)
Output	3	-	-	3	CIELAB prediction (linear regression)

**Table 4:** Ablation study results for the color prediction model.

Model Configuration	$\Delta E_{00}$ (Mean ± SD)	Color Accuracy (%)	SSIM
Full Model	0.52 ± 0.18	98.7	0.9945
Without Physics-Constrained Loss	0.71 ± 0.25	96.8	0.9908
Without Bayesian Optimization	0.63 ± 0.21	97.9	0.9928

**Table 5:** Specifications and validation metrics for imaging system components.

Component	Specification	Operating Parameters	Validation Metrics
Spectrophotometer	X-Rite i1 Pro 2	380–730 nm,10 nm intervals	±0.1 nm precision
Primary Light Source	D50	5003 K ± 50 k	Calibrated daily
UV Light Source	UV-LED	365 ± 5 nm	Power stability: ±1%
Camera System	Panasonic S1R	ISO 100, f/8, 1/60 s	SNR > 40 dB
Geometric Configuration	45°/0°	Fixed position	Angular precision: ±1°
Dataset	70%-15%-15%	Train–Validate–Test	Stratified sampling
Sample Size	1500	ISO standard charts	Coverage: 98% gamut

**Table 6:** Comparative performance of color prediction models.

Model	$\Delta E_{00}$ (Mean ± SD)	Processing Time (s)	Color Gamut Coverage (%)
Polynomial Regression	3.2 ± 10.5	0.12	84.6
CNN	0.95 ± 0.28	0.07	95.2
Inception-Resnet-v2	0.85± 0.15	0.05	96.7

**Table 7:** Model performance under different UV sources.

UV Source	Wavelength (nm)	CNN ΔE (Mean ± SD)	I-ResNet-v2 CNN ΔE (Mean ± SD)	Significance
UVA	365 ± 5	0.8 ± 0.2	0.75 ± 0.1	*p* < 0.001
UVB	310 ± 5	0.85 ± 0.14	0.78 ± 0.11	*p* < 0.001
UVC	254 ± 5	0.82 ± 0.13	0.76 ± 0.1	*p* < 0.001

## Data Availability

The data presented in this study are available upon reasonable request from the corresponding author.

## Appendix A

**Algorithm A1** Polynomial regression with least squares fitting
**Input:** • Color sample set $S=\left\{\left(R_{i},G_{i},B_{i},L_{i},a_{i},b_{i}\right)\right\}\in R^{N\times 6}$, where *N* denotes the number of samples. • Polynomial models set $P=\{\rho_{j}|j\in \left[1,M\right]\}$, where *M* denotes the number of polynomial models. • Maximum polynomial degree $d\in N^{+}$, where ‘+’ denotes a positive integer. **Output:** • Set of M transformation matrices M; • Set of *M* average color differences $\Delta E=\{\Delta E_{j}|j\in \left[1,M\right]\}$. 3. Procedure: a. Initialize empty sets M (for transformation matrices ) and ΔE (for color differences). b. For j = 1 to *M*, perform the following (process each polynomial model): - Construct Feature Matrix: R; - Construct Target Matrix: $H=\left[L^{*}\;\;a^{*}\;\;b^{*}\right]\in R^{N\times 3}$; - Compute Transformation Matrix: $M_{j}={\left(R^{T}R+\lambda I\right)}^{-1}R^{T}H\in R^{n_{j}\times 3}$; - Predict LAB Values: $L^{*}a^{*}b_{pred}^{*}={RM}_{j}\in R^{N\times 3}$; - Compute Average Color Difference $\Delta E_{j}$. c. Return M and ΔE.

## Appendix B

**Algorithm A2** Attention module for feature enhancement
**Input:** Feature map X∈R(C×H×W) Reduction ratio $r\in N^{*}$ **Output:** Enhanced feature map $X^{\prime }\in R\left(C\times H\times W\right)$ 1. Spatial Dimension Reduction: By performing Global Average Pooling (GAP), the spatial dimensions can be reduced from H×W to 1×1 as follows: z=GAP(X)∈R(C×1×1), where GAP(.) denotes the global average pooling operation. 2. Dimensionality Reduction: $z_{1}=\delta \left(w_{1}\;\;z\right)\in R$(C×1×1), where $w_{1}\in R$(C/(r×C)) is a learnable weight matrix and δ(.) denotes the rectified linear unit (ReLU) activation function. The dimension is then restored from C/r back to *C* after performing the dimensionality reduction process. 3. Dimensionality Restoration: $z_{2}=\delta \left(w_{2}\;\;z_{1}\right)\in R$(C×1×1), where $w_{2}\in R$(C×C/r) is another learnable weight matrix. The number of units in the dense layer is then reduced from *C* to C/r after the dimensionality restoration process. 4. Attention Weights Computation: $a=\sigma (z_{2})\in R$(C×1×1), where σ(.) denotes the sigmoid activation function and $\sigma \left(x\right)=\left(1+e^{-x}\right)$. 5. Feature Recalibration: $X^{\prime }=X⊙a$, where ⊙ denotes channel-wise multiplication (each channel in *X* is multiplied by its corresponding attention weight in a). 6. Return $X^{\prime }$

## Appendix C

**Algorithm A3** Bayesian optimization for objective function minimization
**Input:** Objective function f(x): $R^{d}\to R$ Search space $\Omega \subseteq R^{d}$ Maximum number of iterations $T_{max}\in N^{*}$ Initial sampling strategy $S:\Omega \to R^{N}\times R^{N}$ **Output:** Estimated global optimum $x^{*}\to \Omega$ Estimated minimum function value $f^{*}\to R$ 1. Initialize: $D_{0}\leftarrow S\left(\Omega \right)$, where $D_{0}=\left\{\left(x_{i},y_{i}\right),y_{i}=f\left(x_{i}\right),i=1,\ldots ,N\right\}$ 2. **for** t = 1 to Tmax **do** Fit probabilistic surrogate model: $M_{t}\to GP\left\{D(t-1)\right\}$ Compute acquisition function: $acquf\left(x\right)\leftarrow A\left\{(M_{t},x)\right\}$ Determine next evaluation point: $x_{t}\leftarrow \mathrm{argmax}\left\{x\to \Omega \right\}\;\;acquf\left(x\right)$ Evaluate objective function: $y_{t}\leftarrow f\left(x_{t}\right)$ Update observation set: $D_{t}\leftarrow D\left\{t-1\right\}\cup \left\{(x_{t},y_{t})\right\}$ **if** $y_{t}<f^{*}$ **then** $x^{*}\leftarrow x_{t}$, $f^{*}\leftarrow y_{t}$ **end if** **end for** 3. Return $x^{*},f^{*}$

## Appendix D

**Algorithm A4** Design of physical constraint loss function
**Input:** True values $Y=\left\{y_{1},y_{2},\ldots ,y_{n}\right\}\in R^{n}$ Predicted values $\hat{Y}=\left\{\hat{y_{1}},\hat{y_{2}},\ldots ,\hat{y_{n}}\right\}\in R^{n}$ Fluorescence intensity $F=\left\{f_{1},f_{2},\ldots ,f_{n}\right\}\in R^{n}$ Spectral overlap $S=\left\{s_{1},s_{2},\ldots ,s_{n}\right\}\in R^{n}$ Energy conservation $E=\left\{e_{1},e_{2},\ldots ,e_{n}\right\}\in R^{n}$ Model parameters $\theta =\left\{\theta_{1},\theta_{2},\ldots ,\theta_{m}\right\}\in R^{m}$ Hyperparameters $\lambda_{1},\lambda_{2},\lambda_{3},\lambda_{4}\in R_{+}$ **Output:** Total loss L∈R Initialize $L\leftarrow 0,\;L_{color}\leftarrow 0,L_{fluor}\leftarrow 0,\;L_{spec}\leftarrow 0,\;L_{energy}\leftarrow 0,$ $L_{reg}\leftarrow 0$ 1. Calculate color difference loss: **for** *i* = 1 to *n* **do** $L_{color}\leftarrow L_{color}+{\left(y_{i}-\hat{y_{i}}\right)}^{2}$ **end for** $L_{color}\leftarrow \left(L_{color}/n\right)$ 2. Calculate fluorescence intensity constraint loss: **for** *i* = 1 to *n* **do** $L_{fluor}\leftarrow L_{fluor}+{\left(f_{i}-\hat{f_{i}}\right)}^{2}$ **end for** $L_{fluor}\leftarrow L_{fluor}/n$ 3. Calculate spectral overlap constraint loss: **for** *i* = 1 to *n* **do** $L_{spec}\leftarrow L_{spec}+\left(s_{i}-\hat{s_{i}}(\theta \right){)}^{2}$ **end** $L_{spec}\leftarrow L_{spec}/n$ 4. Calculate energy conservation constraint loss: **for** *i* = 1 to *n* **do** $L_{energy}\leftarrow L_{energy}+\left(e_{i}-\hat{e_{i}}(\theta \right){)}^{2}$ **end** $L_{energy}\leftarrow L_{energy}/n$ 5. Calculate regularization term: **for** *j* = 1 to *m* **do** $L_{reg}\leftarrow L_{reg}+{\left(\theta_{j}\right)}^{2}$ **end** 6. Compute total loss: $L\leftarrow L_{color}+\lambda_{2}L_{fluor}+\lambda_{2}L_{spec}+\lambda_{3}L_{energy}+\lambda_{4}L_{reg}$ 7. Return *L*

## Appendix E

**Algorithm A5** High-precision data pre-processing function
**Input:** Raw spectral data D=1 Image set I=1 **Output:** Processed dataset *P* Validation metrics *V* 1. **Initialization:** *P* ←0 *V*← accuracy: [], precision: [], recall: [] 2. **For Each Pair:** (d,I)∈(D,I) 3. **Spectral Calibration:** $d^{\prime }$← Calibration Matrix×*d* Validate Spectral Quality $\left(d^{\prime }\right)$ 4. **Image Processing:** $I^{\prime }$← Registration (I) $I^{\prime \prime }$← Noise Reduction $\left(I^{\prime }\right)$ Validate Image Quality $\left(I^{\prime \prime }\right)$ 5. **Feature Extraction:** *f*← Extract Color Features ($d^{\prime }$, $I^{\prime \prime }$) Validate Features (*f*) 6. **Quality Assessment:** if Quality Metrix (*f*) > threshold, *P*←*P*⋃*f* Update Validation Metrics (***V*)** 7. **Statistical Validation** 8. **Compute Confidence Intervals:** ** $CI=\bar{x}\pm t_{\left(\alpha /2,n-1\right)}\times s/\sqrt{n}$ ** 9. **Outlier Detection Using *z*-score:** ** z=(x−μ)÷σ ** 10. **Quality Metrics:** ** Precision=TP/(TP+FP) ** ** Recall=T/(TP+FN) ** ** F1−Score=2×(Precision×Recall)/(Precision+Recall) **
